# Exploring women’s perceptions of pain when breastfeeding using online forums

**DOI:** 10.1186/s13006-021-00426-9

**Published:** 2021-10-18

**Authors:** Line Caes, Katie Abbott, Sinéad Currie

**Affiliations:** Division of Psychology, Faculty of Natural Sciences, University of Stirling, Stirling, Scotland

**Keywords:** Breastfeeding, Pain, Discomfort, Experiences, Support, Online forums

## Abstract

**Background:**

Despite numerous benefits, many mothers stop breastfeeding soon after birth. A common reason for this is the experience of pain or discomfort. One resource which women use to share their breastfeeding challenges and seek support are online forums. This study aimed to collect data from online forums to explore 1) usage of forums as social support for breastfeeding-related pain; 2) experiences of breastfeeding-related pain; 3) perceptions and strategies to deal with breastfeeding-related pain; and 4) the impact of pain on breastfeeding duration.

**Methods:**

Data was gathered through searches of online forums based in the UK and USA: Netmums, What to Expect and Mumsnet using key terms: ‘*painful breastfeeding’* and *‘sore breastfeeding’*. Data extraction took place in July 2018 and included posts made between 2012 and 2018. Data included 123 posts and 193 replies, analysed using thematic analysis.

**Results:**

The first theme identified was ‘*variation in types of pain’*, highlighting the variety of painful experiences and their descriptions. In particular, this theme reveals the large variety of different types of pain women experience at different stages throughout their breastfeeding journey, as well as the different pain characteristic they focus on in the description of their experience (i.e., location, sensory or physical aspects). Secondly, the theme ‘*perceived causes and explanations for pain*’ revealed how women interpret pain experiences either due to a recognised condition or behavioural cause. The third theme ‘*cessation of breastfeeding related to pain’* identified. How women experience both physical and psychological struggles (e.g., guilt) related to breastfeeding cessation, with pain being a main factor in considering cessation of breastfeeding. Finally, the theme ‘*shared experiences and support*’ identified women’s strong need for both practical and emotional support to deal with pain. Many women look for this support through the knowledge exchange offered on the online forums.

**Conclusions:**

Pain was a key reason for breastfeeding cessation, commonly associated with strong feelings of guilt. The online forums provide a unique form of social support for breastfeeding women to find ways to cope with the pain, while highlighting the urgent need for more appropriate antenatal education on realistic expectation surrounding breastfeeding.

## Background

It is well recognised that breastfeeding is beneficial for infants and mothers. Despite the benefits of breastfeeding, literature from many European countries indicates low initiation and duration rates which often fall short of World Health Organisation recommendations, mainly exclusive breastfeeding for 6 months after birth [1, 2]. One commonly cited barrier to longer breastfeeding duration is nipple and breast pain [3–5]. The experience of pain associated with breastfeeding can differ between individuals, from intolerable pain to mild discomfort [6, 7]. However, this unappealing feeling, no matter how extreme, can be highly detrimental to the breastfeeding experience. For instance, experiences of pain have been strongly linked with breastfeeding cessation [8] and this pain-associated cessation is linked to feelings of guilt, dissatisfaction, upset and risk of postnatal depression [7]. Even in the most determined mothers who have a strong intention to breastfeed, pain is cited as a key reason for cessation [9].

Whilst pain is a common factor experienced during early days of breastfeeding, the underlying reasons for pain and how women manage this experience are varied [10] and not well understood [11]. Literature is also conflicting. Nipple-pain has been attributed to incorrect positioning of the baby [12], however, some evidence refutes this link [6], suggesting that there may be additional physiological or psychological reasons why women commonly experience nipple pain [13]. Furthermore, research has mostly focused on the experience of nipple pain, with little known about the specific type or range of pain women experience when breastfeeding.

It is important to consider how women can be supported to manage or cope with their experience of pain whilst breastfeeding. Qualitative research indicates that women express a mismatch between their general expectations and realities of breastfeeding and a lack of support for emotional needs [8, 14, 15]. This mismatch of expectations and realities has been linked to a woman’s confidence, breastfeeding knowledge and social environment, hence past experience of breastfeeding and support from those around them are key components to meeting breastfeeding expectations and continuing with breastfeeding when presented with setbacks such as pain [16]. There is particularly little exploration of what women expect in relation to pain during breastfeeding or exactly how others influence coping with breastfeeding pain [17]. Furthermore, women actively use online social media to source breastfeeding information and support [18, 19].

The aim of the current research is to utilise online chat forums to explore 1) women’s use of online forum for seeking social support regarding breastfeeding pain; 2) women’s experiences of breastfeeding pain; 3) women’s perceptions and strategies for dealing with breastfeeding pain; and 4) how pain influence women’s decision to (dis)continue breastfeeding.

## Methods

### Participants

Google was used to search for websites which contain discussion forums in which individuals can freely post any comments related to breastfeeding. Websites were found using the following search terms: ‘parents online forums’, ‘parenting forums similar to Netmums’ and ‘most popular parenting forums’. Three websites with discussion forums were selected and used for data collection. These were the three most common websites to appear from the google search. Two were UK based; Netmums [20] and Mumsnet [21] and one was USA based; What to Expect [22]. The three websites were chosen based on their diversity (UK and USA) and popularity as each website had a range of forums with a high quantity of relevant posts. On all included website discussion forums, any individual can join and make a post which is visible to anyone accessing the forum. All three websites administration/customer service teams were contacted via email in order to seek permission to anonymise and analyse the data available on the chat and discussion pages.

### Identifying relevant posts from discussion forums

Word searches were conducted on each of the website discussion forums using the keywords *‘Pain’, ‘Breastfeeding’, ‘Painful Breastfeeding’* and *‘Sore Breastfeeding’*. These searches resulted in the identification of posts which contained the keyword searched for. Word searches of discussion forums were conducted in July 2018 in Stirling, Scotland by one researcher (KA).

### Measures

Each post identified from the word searches were read in the order they were displayed. Inclusion criteria for a post to be included in analysis was (1) written in English, (2) focuses on challenges related to painful breastfeeding and (3) written by the individual explaining their experience. All identified posts were read thoroughly to ensure they met these criteria. All replies to the post were read and included in analysis.

A total of 123 posts were identified from the word searches, which were made between 2012 and 2018. All 123 posts were eligible for inclusion and analysis. A total 193 reply comments accompanied the posts, resulting in a total of 316 data extracts included in the analysis. This included 146 data extracts from What to Expect, 89 from Mumsnet and 81 from Netmums.

One research assistant (KA) conducted the data extraction of eligible posts (2012–2018) in July 2018. The information extracted included 1) the link for the post on the website, 2) whether a post was original or a reply, 3) the date of which the post was submitted to the forum, 4) full text of the post.

### Analysis

The secondary analysis of online data from discussion forums was conducted using thematic analysis [23]. As no pre-existing framework on breastfeeding pain is available, the stages of inductive thematic analysis were followed to analyse all selected posts to answer the research questions. Firstly, in order to familiarise with the data, the researcher read through all posts in detail numerous times, at this stage no interpretations were made. Secondly, to generate a better understanding of the data, the research assistant coded the data into related groupings. This resulted in generation of 25 codes. Thirdly, both the senior authors (SC and LC) and the researcher analysed the codes and as a team produced four main themes together. Fourthly, the researcher reviewed all the data extracts using the four identified themes to ensure that the themes were fully representative of the data. Fifthly, after generating a thematic map of the data, both senior authors and the researcher defined and named the themes and finally key quotes were selected to best represent each theme.

## Results

The inductive thematic analyses generated four themes and a range of sub-themes (see Table 1).

**Table 1:** Themes, sub-themes and descriptions of the data extracted

Theme	Description	Sub-themes
Variation in types of pain	Different types of pain that women experienced and referred to throughout their experience of breastfeeding	- Pain location - Sensory and emotional aspect of pain - Physical process associated with the pain
Perceived causes and explanations for pain	Interpretations and reasons why women may be experiencing different types of pain	- Recognised condition - Behavioural explanations
Cessation of breastfeeding related to pain	Reasons and struggles, related to pain, why women consider stopping breastfeeding	- Physical aspects - Psychological aspects
Shared experiences and support	Types of support, experiences and knowledge that women provide to support other women on their breastfeeding journey	- Sharing experiences - Practical support - Experience and support from health professionals

### Variation in types of pain

The first theme generated from the data was ‘variation in types of pain’. This theme represents the large variety of different types of pain women explain and experience at different stages throughout their breastfeeding journey. Furthermore, the various ways in which women describe these pain experiences highlights how women differ in the way they describe pain, ranging from descriptions on 1) the pain location, 2) the sensory or emotional characteristics of pain and 3) the physical process the pain is associated with. Across these three types of descriptions, a total of seven distinct types of pain women experience in relation to breastfeeding could be identified. These types of pain were sore nipples, cracked nipples, let down pain, painful breasts, painful latch, sharp pains and unexpected pains.

With respect to descriptions focussed on pain location, a large number of posts mentioned the experience of painful nipples, with some posts referring to the general feeling of sensitive or sore nipples while others refer to the more specific experience of cracked nipples:

*‘My nipples have been blistered, I’ve been using cream and when not using that using nipple healing cups. One nipple is still really sore but seems to be healing and the other is still a bit sore.’* (P1 Mumsnet, post).

*‘I am cracked, bleeding, blistered and even wet myself yesterday with the pain of it. I am alternating between biting a towel stamping my feet and making moaning noises.’* (P2 Netmums, post).

However, for a substantial number of women the experience of pain is not limited to the nipples and encompasses the entire breast, even without any specific nipple damage.

*‘for almost a week now I’ve had really sore breasts, particularly in the evening to the point! There is no damage to my nipples, the pain is in my actual breasts. They are hot and the pain is like a burning pain.’* (P3 Netmums, post).

In terms of the sensory aspects of the pain associated with breastfeeding, women often described the experience as ‘sharp shooting pain’ that could be felt in the breast but also the larger area around the breast, including the shoulders. For some women, this description also had an emotional layer as they experienced the pain occurring randomly and unexpected, especially when the pain occurred months after they have been breastfeeding. Such an unexpected occurrence of pain could be induced by unrealistic expectations women posit around breastfeeding.

‘*sharp shooting pain in my shoulder blade behind the breast the is baby feeding on.’* (P4 Netmums, post).

*‘So I have been breastfeeding for 8 months and randomly (when I’m not nursing) I get a shooting sharp pain in my breast . . . any clue what it could be??’* (P5 What to Expect, post).

Lastly, women also described their pain in terms of the physical process it was related to, with the two most common processes being ‘let down’ and ‘latch’. Let down pain relates to a type of pain that occurs when milk is beginning to flow and typically occurs in the first few weeks of breastfeeding.

*‘Just wondering if you had let down pain when breastfeeding and if so for how long, my son is 3 weeks old today and the pain at initial latch on is excruciating.’* (P6 Netmums, post).

A painful latch is pain related to how the baby latches on to the nipple and occurs when the baby does not properly latch onto the mother’s breast.

*‘Sometimes, (mostly on the ‘bad’ side) it can be absolutely excruciating for the whole feed.’* (P7 Mumsnet, post)’.

### Perceived causes and explanations for pain

The second theme focuses on women’s interpretations and perceptions of pain during breastfeeding as well as the interpretations and perceptions given by other users who are replying to the original post. In line with the commonly observed application of the biopsychosocial model of pain which primarily focusses on biological and psychological causes of pain experiences and largely ignoring the contribution of social factors (Craig, 2018), the perceived causes and explanations for breastfeeding pain experiences can be divided into ‘recognised conditions’ (i.e., biological explanation) and ‘behavioural explanations’ (i.e., psychological explanation).

The first sub-theme, entitled recognised conditions, includes conditions such as mastitis, thrush, Raynaud’s syndrome or tongue tie, which are all known to be associated with pain. Many women spoke of their familiarity with these recognised conditions, where they commented on their personal experience:

*‘Recently we have had mastitis, it is painful and I came very close to stopping feeding him as I was just in tears everytime I fed him.’* (P8 Netmums, reply).

The posts went beyond providing suggestions on the causes, with some women also giving advice on how they dealt with the condition they were faced with:

*‘It could be thrush! I took her to the doctor and he saw some hidden patches behind her gums. I rub a gel in her mouth and on my nipples twice a day and it seems to be helping.’* (P9 What to Expect, reply).

The second sub-theme reflects the behavioural explanations defined as any behavioural cause, reason or solution given for pain during breastfeeding. An example being various ways to clear blocked ducts which can cause pain. Most of the suggestions for behavioural causes came from other users replying to original comments made by mothers looking for help and advice about certain types of pain. Similar to the post providing a recognised condition as an explanation for the pain, these posts often went beyond describing the cause and also provided potential ways of coping with the pain:

*‘Sounds like a clogged duct. Use a hot compression before you feed or pump. Apply coconut oil and massage the area while baby nurses or you pump. Push on area that feels hard/hot and push towards nipple. When you shower use the hottest water you can and massage some more.’* (P10 What to Expect, reply).

*‘the attachment and positioning will most probably be the problem for the pain when latching on. Def ask hv [health visitor] to watch you when you feed. I always tell mums to remember: Tummy to Mummy, (babies) Nose to Nipple and keep the head and body in a straight line’.* (P11 Netmums, reply).

### Cessation of breastfeeding related to pain

The third theme, ‘cessation of breastfeeding related to pain’ encompasses the pain related reasons, struggles and psychological consequences women mention in relation to stopping, or considering stopping, breastfeeding. Similar to the second theme, the identified struggles women report on pain-related breastfeeding cessation can be divided into physical and psychological aspects.

Physical reasons and struggles mainly referred to the experience of breastfeeding being extremely painful in the first few weeks due to breasts and nipples adapting to feeding. Women replying to cessation-related comments, comments where women were considering stopping breastfeeding due to pain, within original posts made by others often provided comfort to other women by reassuring that the pain they are experiencing may not necessarily be caused by something they are doing wrong, or any medical conditions but instead just because their body is adapting to the change it is facing.

*‘Your nipples are tender at first so, imagine going from not having anything touching your nipples, to having a baby latched on every two hours for 30 minutes or more at a time WHILE your nipples are already tender and hurting from the hormones of having a baby.’* (P12 What to expect, reply).

A common, recurring theme across all forums reflected the psychological struggles or consequences women experience when considering breastfeeding cessation, with a considerable number of women reporting feeling guilty:

*‘I feel a total failure on this, and I don’t want to give up, but it’s being so hard and painful. I also feel guilty because I find myself wishing it to end soon, I mean wishing the time going faster until the point that she’s 2 years and I can stop breastfeeding and I feel like such a terrible mom.’* (P13 What to Expect, post).

The psychological side of breastfeeding indicates how difficult it can be for mothers to cope with the pain they are facing and how they experience feelings of guilt or sadness about possibly quitting breastfeeding earlier than recommend, as they know it is beneficial for their baby. A commonly reported strategy to overcome feelings of guilt around ceasing feeding at the breast, while managing the pain was the use of breast pumps. Indeed, many women reported making use of breast pumps as a compromise to reduce the pain and continue providing the baby with breast milk without having to endure the pain of physically breastfeeding, which helps them to not feel guilty about providing their child with breast milk:

‘*If you are in pain, you can pump or hand express to relieve the pressure.’* (P14 Mumsnet, reply).

### Shared experiences and support

The final theme ‘shared experiences and support’ represents the range of support and knowledge exchange that women offer throughout the forums. The sub-themes include sharing experiences; practical support and experience and; support from health professionals.

Many mothers offer, through the replies, their own experiences of breastfeeding in order to help other women and provide emotional support:

*‘Most importantly is your well being – healthy Mum = healthy baby no matter how she is fed, it makes no difference, you can’t look at a room of adults and tell who was BFd [breastfed] and who wasn’t. Please seek help if you wish, or continue with formula and relax and enjoy this precious gift of a child without guilt. Sending unmumsnetty hugs. Also remember you’re under massive hormonal influences at the moment and so everything will be feeling magnified.’* (P15 Mumsnet, reply).

From the data, it is evident that many women who post an original post are not looking for specific reasons of why they are experiencing pain but instead they are looking for emotional support and to know that other women have faced similar experiences to them.

In addition to emotional support, many women also offer each other practical support for reducing pain. This includes recommending a range of medications or creams or offering advice on different latching techniques, this support is shown in the following quotation:

*‘Aim you nipple to the back of babies mouth (in most cases this is enough to get enough in for a good latch, as the pink area will go in also 2). If you still have pain, take baby off by using your little finger next to your nipple so baby looses grip . . . and try again until you have minimum of pain ( I cant say completely no pain right now, as you nipples are sore), but the pain you will very minimum, if nothing at all, and if it is correct, no pain at all within a few days.’* (P16 Netmums, reply).

Finally, this theme covers the mothers’ perceptions of health professionals and their ability to deal with pain during breastfeeding and offering guidance and support to women. Many women have different opinions on health professional’s ability to deal with the problem. For example, some women felt that midwives and other professionals offer false hope in saying breastfeeding should be a pain-free experience:

‘*I am baffled by the unrealistic advice given to pregnant women about breastfeeding by health professionals- If it hurts you are doing it wrong. 99% of women can successfully breastfeed. I understand their role is to encourage women to do it but surely being honest about the difficulties of starting that many women encounter would be more helpful. Mothers who then have difficulty are left feeling they’ve failed when they are trying to pick up the pieces. Likewise problems may not get as bad if we were honest to women about it.’* (P17 Mumsnet, post).

On the other hand, some women reported positive experiences with different health professionals who helped with advice and support through their difficult time of breastfeeding, for example one user had a positive experience with their midwives:

*‘Have been seeing midwives everyday for extra support which is fab and have now been referred to the breast feeding expert and also GET THIS been referred to see the TONGUE TIE consultant!’* (P18 Netmums, reply).

Both of these quotations reflect the different experiences that mothers can have with different health professionals, highlighting the importance of having good training in place for these professionals to be able to recognise and deal with, different types of medical conditions or behaviours that can cause mothers to have a bad experience with breastfeeding.

## Discussion

Using thematic analysis to gain an insight into the pain women experience during breastfeeding and how online forums are being used to share experiences, four main themes were identified: variation in types of pain, perceived causes and explanations for pain, cessation of breastfeeding related to pain; and shared experiences and support. These findings extend the findings of a recent qualitative study where pain was recognised as a main reason for early breastfeeding cessation [8]. Similar to the stories from the online forums, these women [8] reported varying experiences of support from healthcare professionals, with most receiving inadequate information. Our findings expand on this knowledge by providing a more detailed insight in the various experiences of pain and associated perceived causes, as well as the importance of online forums for seeking and receiving social support.

Our findings complement previous literature which has revealed the prevalence of pain experiences during breastfeeding, with nipple pain being the most commonly reported type [6, 7, 14]. Our findings indicated a large variety of pain experiences, not just nipple pain hence a better understanding of the various types of pain experienced during breastfeeding is critical in designing appropriate interventions to manage the pain experience. Existing intervention development has mainly focused on the experience of nipple pain [19], which might not be appropriate for other common pain experiences, such as painful latch or let down pain.

Gaining such detailed understanding of women’s experiences might not have been possible using more traditional research methods, which face challenges like selection bias [24]. Online discussion forums have the ability to reach a larger number and variety of people and lack the steering by the researcher’s interests/orientation. Consequently, online forums allow users to share and attain real-life experiences and, therefore, set realistic expectation about the potential pain, and underlying cause, that can be experienced during breastfeeding. However, not much research has been conducted concerning ungoverned online forums, therefore, further investigations is needed to evaluate the potential impact for helping users to cope with pain and breastfeeding.

Findings point to the two common aspects within the user’s experiences that deserve more attention, that is the role of peer support and setting realistic expectations of breastfeeding. These findings link in well with existing qualitative work which has revealed the need for realistic, not idealistic, expectations for breastfeeding [25]. These authors also highlight how social support groups can help to normalise breastfeeding experiences [25]. A recent qualitative study also indicated that online breastfeeding support (including forums) was reassuring, empathetic and more accessible for women compared to traditional sources of breastfeeding support [26]. Indeed, our findings revealed that receiving emotional support from other users is common in online forums. This emotional support is linked to the psychological side of breastfeeding struggles, such as feelings of guilt. As found in previous research [8, 27], it is common for women to feel guilty for breastfeeding cessation. This strong prevalence of guilt highlights how women are aware of and convinced by the benefits of breastfeeding, which may suggest that information provision surrounding the benefits of breastfeeding may not actually be an effective strategy to support breastfeeding continuation.

Consequently, the findings indicate that psychological support, such as peer support, is crucial in overcoming the psychological impact of pain experiences. An important aspect of this peer support observed in our data was aimed at providing reassurance, for example it is okay to use breast pumps as a form of respite for the mother. Peer support is a unique type of support that women can give each other that goes beyond the care and support received within a formal healthcare setting [28] and strongly reduces the feelings of isolation. Although it needs to be acknowledged that the support provided by peers might not always be accurate and credibility of the sources needs to be considered carefully [29], the availability of peer support outside the healthcare system could reduce the reliance on healthcare professionals as a first port of call [30]. While further research is needed to establish the impact of peer support, specifically for breastfeeding pain [31], the beneficial impact of peer support on individual’s pain self-management confidence and reduced experiences of distress is well-established for chronic pain [32].

Beyond providing normalisation, peer support can also assist women in setting realistic expectations. Our findings indicate how many forum users were unaware that the experience of pain during breastfeeding is common. Many forum users who replied to original posts advised that it does in fact take a few weeks for your breasts to adapt to breastfeeding, as it is a new experience for both mother and baby. Similar to the benefits of including realistic expectation setting within antenatal interventions to reduce anxiety and depression in new mothers [33], setting realistic expectations around breastfeeding pain may be beneficial for both antenatal and postnatal women.

The relevance to set realistic expectations for breastfeeding and potential pain experiences also came to light in forum users’ mixed views on the helpfulness of the health professionals and their ability to provide relevant care. Some users felt health professionals did not focus enough on breastfeeding being painful, so it came as a surprise when it was painful and subsequently increased the worry for the women and their likelihood of quitting. On the other hand, several users did comment on their positive experiences with health professionals, giving them credit for being supportive and getting to the bottom of their issue. Raising awareness of breastfeeding pain requires optimal communication skills and hence these mixed experiences with healthcare professionals highlight the need to provide healthcare professionals with appropriate support on how to convey the normal experience of pain during breastfeeding without scaring mothers and thereby jeopardizing either the start or continuation of breastfeeding. It also relies on women feeling comfortable and confident to contact health professionals for help or advice as soon as possible.

### Strengths and limitations

This study applies a novel, naturalistic methodology to analyse data from existing, public online forums to explore what women really experience in terms of breastfeeding associated pain. The findings show the range of types of pain experiences, the struggles women face and the support given by other forum users. It sheds light on the difficulties many women face when breastfeeding, both physiological and psychological. However, our findings need to be considered in light of some limitations. Although online forums provide a rich and varied amount of lived experience, there is no information available on the demographics of the forum users. Consequently, mothers who use online forum might not be representative of the entire population. Furthermore, while the posts typically provided detailed descriptions of women’s experiences, this medium does not allow for exploration of relevant aspects of the experiences in further detail. In addition, with the rise of social media use over the past decade, other online sources such as Facebook and Instagram may be avenues for women to seek help, advice and support. The current study did not explore these social media outlets. Further research is needed to explore the impact of online forums and other social media outlets on women’s experiences and breastfeeding continuation as well as the underlying mechanisms of such benefit.

## Conclusions

Our findings have relevant clinical implications. In order to increase the initiation and duration of breastfeeding, interventions should set realistic expectations on the common types of pain women may experience. These interventions should bring a focus to the different types and causes for pain such as thrush, or mastitis and what self-management techniques can be implemented to prevent or reduce the pain. Furthermore, raising awareness of when to seek help for pain experiences, is also crucial. In line with interventions for other types of acute pain, it is important to address the psychological struggles. In particular, the findings reveal that it is important to make women aware of the psychological impacts of pain (i.e., guilt) and are provided with coping mechanisms to overcome these feelings (e.g., cognitive restructuring). Integrating realistic expectations of breastfeeding pain during the antenatal period could help to reduce the worry if or when the women experience pain. Our findings also provide preliminary, but promising, evidence for the potential role of online peer support to encourage sharing breastfeeding struggles and the emotional impact and provide a normalisation, to women who are struggling to keep going.

## Data Availability

The data analysed during the current study are publicly available on the aforementioned online forum sites. In addition, the generated dataset is available from the corresponding author.
